# Smartphone-powered iontophoresis-microneedle array patch for controlled transdermal delivery

**DOI:** 10.1038/s41378-020-00224-z

**Published:** 2020-12-28

**Authors:** Jingbo Yang, Yanjun Li, Rui Ye, Ying Zheng, Xiangling Li, Yuzhen Chen, Xi Xie, Lelun Jiang

**Affiliations:** 1 Guangdong Provincial Key Laboratory of Sensor Technology and Biomedical Instrument, School of Biomedical Engineering, Sun Yat-Sen University, Guangzhou, 510275 China; 2 State Key Laboratory of Optoelectronic Materials and Technologies, School of Electronics and Information Technology, Sun Yat-sen University, Guangzhou, 510275 China

**Keywords:** Engineering, Bionanoelectronics

## Abstract

The incidence rate of diabetes has been increasing every year in nearly all nations and regions. The traditional control of diabetes using transdermal insulin delivery by metal needles is generally associated with pain and potential infections. While microneedle arrays (MAs) have emerged as painless delivery techniques, the integration of MA systems with electronic devices to precisely control drug delivery has rarely been realized. In this study, we developed an iontophoresis-microneedle array patch (IMAP) powered by a portable smartphone for the active and controllable transdermal delivery of insulin. The IMAP in situ integrates iontophoresis and charged nanovesicles into one patch, achieving a one-step drug administration strategy of “penetration, diffusion and iontophoresis”. The MA of the IMAP is first pressed on the skin to create microholes and then is retracted, followed by the iontophoresis delivery of insulin-loaded nanovesicles through these microholes in an electrically controlled manner. This method has synergistically and remarkably enhanced controlled insulin delivery. The amount of insulin can be effectively regulated by the IMAP by applying different current intensities. This in vivo study has demonstrated that the IMAP effectively delivers insulin and produces robust hypoglycemic effects in a type-1 diabetic rat model, with more advanced controllability and efficiency than delivery by a pristine microneedle or iontophoresis. The IMAP system shows high potential for diabetes therapy and the capacity to provide active as well as long-term glycemic regulation without medical staff care.

## Smartphone-powered drug delivery device

A device developed by researchers in China uses a microneedle array to efficiently and painlessly deliver precise doses of insulin and can be used by patients without help from medical staff. The new system was developed by a team led by Xi Xie and Lelun Jiang of Sun Yat-Sen University. The array of microneedles is pressed against the skin to create micro-holes and then automatically retracted. A mild electric current then drives charged nanovesicles carrying insulin through the holes. The circuit is powered and controlled via a smartphone, which can alter the intensity of the current to determine the dosage of insulin delivered. The team showed that the device effectively delivered insulin to a diabetic rat model with no obvious harmful effects. It thus offers a powerful tool for efficient, controllable, and convenient delivery of macromolecular drugs.

## Introduction

Diabetes is an important chronic disease characterized by an inability to regulate the blood glucose level (BGL)^1,2^, and the resulting complications are the main causes of disability and death^3,4^. There are three convenient methods to treat diabetes, namely, oral hypoglycemic drugs, the subcutaneous injection of insulin or an insulin pump, while continual and long-term exogenous insulin is one of the most effective and safe ways for type-1 diabetes therapy^2,5,6^. However, these conventional techniques still suffer from several issues. For example, oral hypoglycemic drugs inevitably cause hepatic first-pass metabolism and gastrointestinal degradation side effects^7,8^. Furthermore, a major constraint of subcutaneous injection and the insulin pump is inadequate safety and comfortability because of the traditional metal needles^9–11^. Thus, such traditional methods are usually painful and lack effective glycemic control, which may lead to a high risk for complications, such as limb amputation and blindness^12,13^. In addition, hypoglycemia can also account for seizures, brain damage, or even death^5,14^.

The microneedle array (MA) technique is an effective and minimally invasive method to realize transdermal drug delivery in a pain-free manner^15,16^. MAs are generally applied on the skin to penetrate the stratum corneum (SC) without stimulating nerves and puncturing blood vessels, thereby delivering drugs across the SC to enhance therapeutic efficacy^17–19^. MA-assisted drug delivery has gained great achievement in the treatment of various diseases. For example, Vora et al.^20^ reported a bilayer MA containing polylactic-coglycolic acid (PLGA) nano-microparticles to provide a promising method for controlled transdermal administration. Furthermore, an MA loaded with dual mineralized particles successfully achieved a feedback diabetes treatment^21^. Ye et al.^14^ developed an MA patch containing ‘glucose-signal amplifiers’ to regulate insulin secretion for the treatment of type-1 diabetes. In addition, MA-mediated delivery of insulin or metformin also yielded satisfactory effects in rat models^1,13,22–24^. While there has been remarkable success through chemical and material routes to control MA-mediated drug delivery, the integration of MA systems with electronic devices to precisely control drug delivery has rarely been realized. The lack of appropriate combinations with electronic control systems limits the development of MA delivery systems for integration with electronic, information and intelligent technologies.

On the other hand, iontophoresis offers an attractive strategy for transdermal drug delivery with accurate control through electric signals^25–27^. This technique employs a mild current to promote charged therapeutic agents across the skin layer and even into the systemic circulation and thus significantly enhances the transdermal delivery of the drug^28,29^. The major advantage of iontophoresis is that the delivery can be achieved with exact controllability of the electrified time and parameters^26^. Gelfuso et al.^30^ evaluated iontophoresis for voriconazole delivery, which demonstrated the capacities of iontophoresis to enhance drug penetration and potency. In addition, it was reported that the amount of ropinirole hydrochloride could be precisely delivered by different current densities, which allowed the customized treatment of Parkinson’s disease^31^. Although iontophoresis has been shown to be an excellent technique for the delivery of small drug molecules (such as methotrexate^32^, diclofenac, and calcein^33,34^), it is challenging for iontophoresis to deliver macromolecules such as proteins due to the barriers of the SC^35,36^. This limitation has led to the low efficiency of iontophoresis and has prompted new strategies in combination with other techniques.

**Fig. 1:**
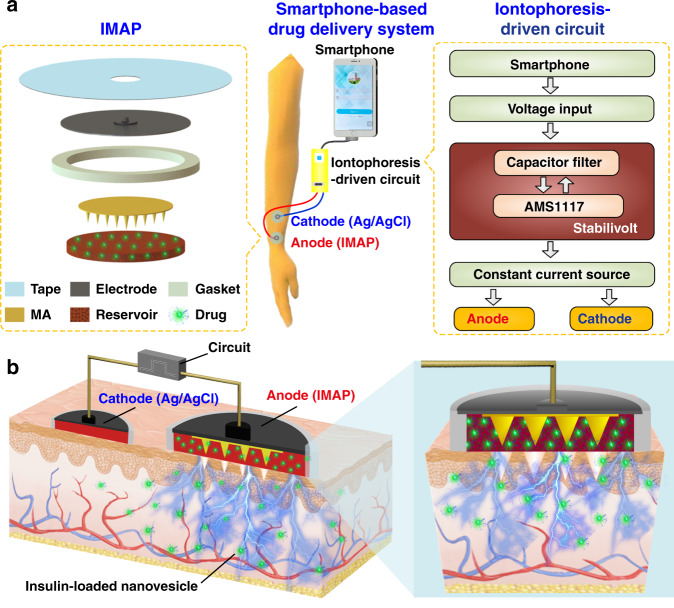
Schematic illustration of the smartphone-powered IMAP drug delivery system and its mechanism. **a** Schematic illustration of a smartphone-powered transdermal drug delivery system. It mainly consists of an IMAP (the expanded view is shown on the left), an iontophoresis-driven circuit (the structure diagram is shown on the right), and a smartphone. The IMAP is composed of a medical tape, an electrode with conductive film, an anti-seepage gasket, an MA and a reservoir loaded with drug solution. The portable circuit can stabilize the input voltage and output a constant current for the iontophoresis of drugs under the power supply of the smartphone. **b** Schematic representation of the drug delivery mechanism of the IMAP: “Penetration, Diffusion and Iontophoresis”. Once the compression on the IMAP is removed, the MA will retract into the reservoir, and microholes are created on the skin. Then, a mild electric current is conducted between a pair of electrodes (IMAP and Ag/AgCl electrode) to drive the charged therapeutic molecules through microholes.

In this work, we present an iontophoresis-microneedle array patch (IMAP), where an MA is first pressed on the skin to create microholes through the SC layer and then is retracted automatically, followed by the iontophoresis delivery of macromolecular drugs through these created microholes in an electrically controlled manner. The MA creates microholes serving as a necessary pathway in order to allow the macromolecules to enter, while coupled iontophoresis actively drives macromolecule delivery in an electrically controllable manner (Fig. 1). Although iontophoresis has been proven to enhance the delivery efficacy of an MA attached to the skin, the initial state of the penetration holes by the MA are still challenging to maintain, and sustained and repeated drug delivery is still difficult to realize. In addition, the permanent penetration of the skin by the MA may facilitate the risk of a skin allergy if the MA stays in the skin for a long time. To address the abovementioned issues, instead of leaving the microneedles permanently attached on the skin, our IMAP employs a different design, where the MA retracts after creating the necessary holes in the skin. The IMAP integrates a solid MA with iontophoresis into a single transdermal patch powered by a portable smartphone, achieving a one-step drug administration approach of “Penetration, Diffusion and Iontophoresis”. Using touch-actuated ‘press and release (P&R)’ actions, the IMAP can easily reopen the self-healing microholes created in the skin for long-lasting passive diffusion and active iontophoresis of the drug solution. To further enhance the iontophoresis performance of the IMAP, insulin was encapsulated in positively charged nanovesicles to improve transdermal drug permeation owing to electroosmosis and electrostatic interactions^27,37^. The IMAP coupled with iontophoresis and nanovesicles was demonstrated to achieve the synergistic and safe enhancement of the in vivo treatment of type 1 diabetic rats. This system exhibits the best BGL control and the effective avoidance of hypoglycemia with the longest normoglycemic state for 6.8 h, which is 3.1 times that in the injection group. With these advantages, our IMAP with a simple drug administration mechanism is a promising alternative for type 1 diabetes treatment that provides both active and long-term glycemic regulation in a safe and controllable manner.

## Results and discussion

### Drug delivery mechanism

A smartphone-powered IMAP drug delivery system was developed for transdermal diffusion and iontophoresis of the drug solution, as shown in Fig. 1a and Video S1. This system consists of a smartphone, an iontophoresis-driven circuit, an Ag/AgCl electrode, and an IMAP. The smartphone is used for powering the iontophoresis-driven circuit via a charging port. The iontophoresis-driven circuit stabilizes the input voltage and outputs a constant current for iontophoresis. The expanded view of the IMAP is presented in Fig. 1a (left). The patch is composed of a medical tape with an electrode and an anti-seepage gasket, an MA and a medical sponge, which are concentrically bonded together. The medical sponge is porous and can be loaded a large amount of drug solution. The IMAP is compressible owing to the good elasticity of the sponge and gasket. The MA can poke the skin and then detach from the skin for transdermal diffusion and iontophoresis of the drug solution.

The transdermal drug administration strategy of the IMAP is “Penetration, Diffusion and Iontophoresis”. (1) Skin penetration by the MA: Since the IMAP is pressed, the MA penetrates the sponge reservoir and disrupts the SC, creating transient aqueous microholes. Once the compression on the IMAP is released, the MA retracts into the medical sponge because of the inherent elastic rebound energy. (2) Passive diffusion: MA-induced microholes are directly exposed to the drug solution stored in the sponge. According to Fick’s law, the drug solution will diffuse into the skin through the aqueous microholes. The velocity of passive diffusion is mainly determined by microholes and drug solution concentration, as illustrated in Fig. S1. (3) Active iontophoresis: a mild electric current is conducted between a pair of electrodes to drive charged therapeutic molecules through microholes into systemic circulation by the predominant driving forces of electromigration and electroosmosis^28,29,35^. The amount of transported therapeutic molecules mainly depends on the charged potential of the molecules, skin barrier impairment by the MA, applied electric field intensity and treatment duration^37–39^. (4) Diffusion and iontophoresis of the charged drug-loaded nanovesicles: Iontophoresis is combined with electropositive nanovesicles to enhance the penetration of the drug-loaded nanovesicles^27,37,40^, as illustrated in Fig. 1b and Video S1. Furthermore, owing to the recovery ability of the skin, the created microholes gradually shrink and self-heal to closure after MA removal^41^. One of the key advantages of this design is that the users can repeat the abovementioned P&R processes to reopen the microholes for a new round of drug administration, resulting in long-lasting positive diffusion and active iontophoresis.

Above all, the smartphone-powered IMAP can achieve four-drug administration strategies, including drug diffusion across intact skin, drug diffusion across poked skin, drug diffusion and iontophoresis across intact skin, and drug diffusion and iontophoresis across poked skin. The administration strategy of drug diffusion across poked skin using a touch-actuated MA patch was reported in our previous work^41^. Compared with the other four administration methods, the insulin-loaded touch-actuated MA patch exhibited the best hypoglycemic effect. In particular, the drug administration strategy of the IMAP using positive diffusion and active iontophoresis of drug-loaded nanovesicles for highly effective drug delivery will be investigated comprehensively in the following sections.

### Fabrication and characterization of the IMAP

**Fig. 2:**
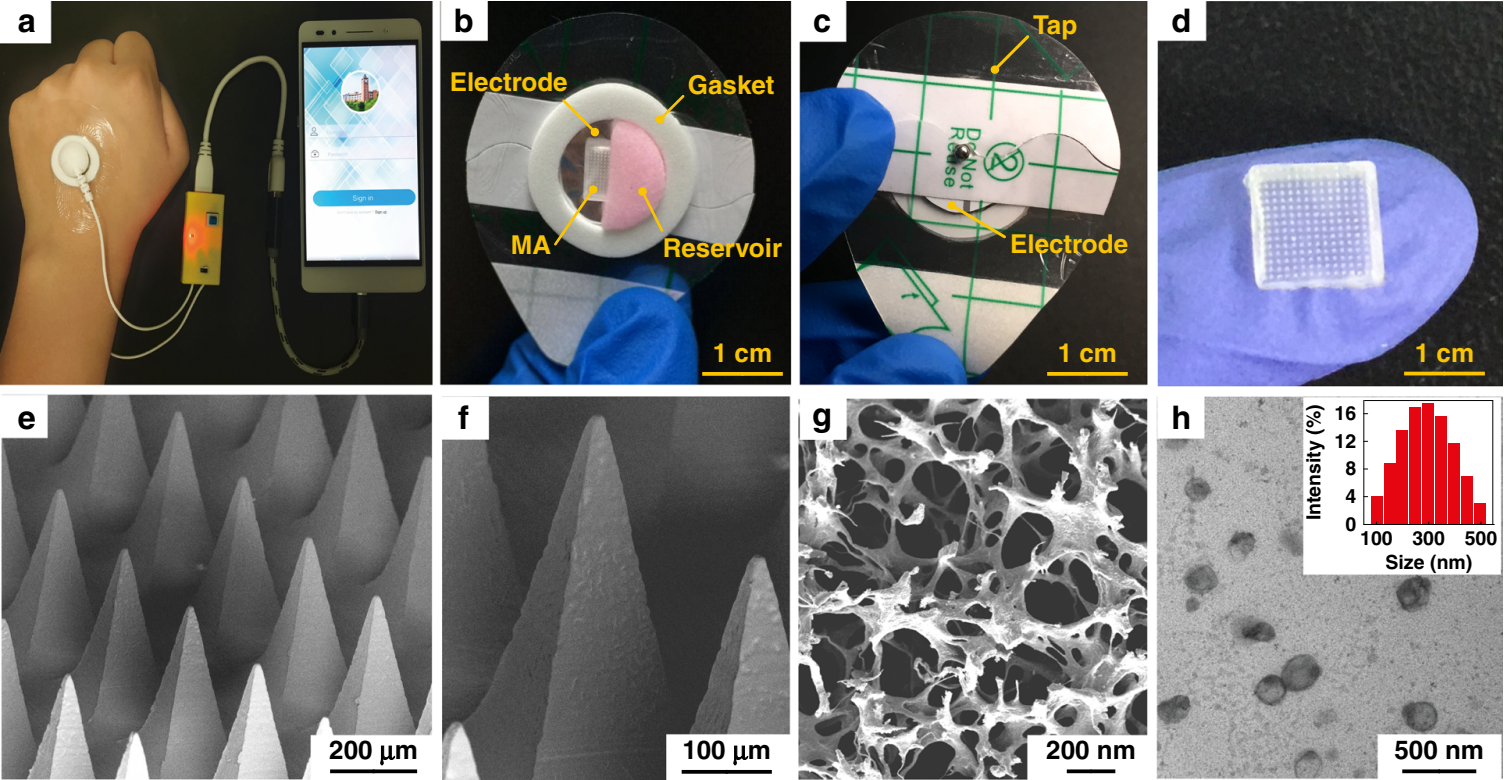
Morphology and composition characterization of the smartphone-powered IMAP drug delivery system. **a** Photo of a smartphone-powered IMAP drug delivery system. The IMAP employed as the anode for iontophoresis is taped on the back of a hand. The iontophoresis-driven circuit was encapsulated in an insulation shell and is directly powered through the smartphone charging port. **b**–**c** The front and back images of the IMAP. The main components, including medical tape with an electrode and an anti-seepage gasket, an MA and a sponge reservoir, are concentrically bonded together. **d** The digital photograph of PMMA MA, which takes up less space than a fingernail. **e** Scanning electron microscope (SEM) image of the MA whose pyramid microneedles are neatly arranged into a square array. **f** The magnified SEM image of a single microneedle that possesses a sharp tip for disrupting the SC structure. **g** SEM image of the sponge, which contains numerous randomly distributed micropores filled with high doses of drug formulations. **h** Transmission electron microscopy (TEM) image of insulin-loaded nanovesicles (insert: the statistical diagram of the size distribution of the insulin-loaded nanovesicles).

According to the above design, the IMAP and the portable smartphone-powered iontophoresis-driven device were developed, as shown in Fig. 2a. The iontophoresis-driven circuit was encapsulated in a 3D printed insulation shell (yellow). The miniature iontophoresis-driven circuit mainly consists of two modules: input voltage stabilization and constant current output (Fig. 1a, right and Fig. S3). The iontophoresis-driven circuit was directly powered through the smartphone charging port. The power supplied by the smartphone was rectified and stabilized using a low dropout voltage regulator (AMS1117, Zhiquan Electronics Fittings Factory, China) and two capacitor filters. The obtained stable voltage was transferred into a constant current output using a circuit of the Wilson current source. The Wilson current source mainly contains three identical triodes (2N 3906B331, Zhiquan Electronics Fittings Factory, China), and the detailed derivation process is shown in Fig. S3c, d of the Supporting Information. The output constant currents of the iontophoresis-driven circuit can be tuned by different equivalent resistances. The iontophoresis-driven circuit can output constant currents of 1, 2, and 3 mA using a switch. According to the above design, a printed circuit board was fabricated (Fig. S3b), and the insulation shell was 3D printed (Fig. S3a). The smartphone-based iontophoresis-driven device is portable for self-administration with a small size (40 mm × 20 mm × 15 mm) and a light weight of only 20 g. The device costs <3 USD to assemble one device, confirming its cost effectiveness. In addition, the device is energy-saving, with several milliwatts of total consumption for iontophoresis. Therefore, the smartphone-powered iontophoresis-driven device is portable and convenient for self-administration in daily life.

The main components of the IMAP, including medical tape with an electrode and an impermeable gasket, an MA and a porous sponge, are demonstrated in the images, as shown in Fig. 2b, c. The tape can closely attach the IMAP on curved skin and minimize drug loss due to its flexibility and waterproof property. The electrode, in direct contact with the drug solution, is employed as an anode for iontophoresis. The elastic anti-seepage gasket can effectively avoid the leakage of the drug solution stored in the sponge during skin penetration. The MA packed in the medical sponge is used for skin penetration to create microholes, allowing transdermal diffusion and iontophoresis of the drug solution. The images of the MA prepared from polymethyl methacrylate (PMMA) with satisfying mechanical strength and good biocompatibility are shown in Fig. 2d–f. Pyramid microneedles are neatly arranged into a 15 × 15 square array. The average height, tip diameter, and base diameter are ~600, 15, and 200 µm, respectively. As exhibited in Fig. 2g, the sponge is porous and contains numerous micropores (porosity: ~95%), and it is employed as the reservoir of the IMAP, which can be filled with large doses of drug formulations. The detailed parameters of the components are listed in Table S1 of the Supporting Information. All materials employed for the assembly of the IMAP components have been widely applied in the clinic and are commercially available. Moreover, IMAP can be easily disposed of as a wound dressing.

Insulin therapy has exhibited the most common and promising therapeutic effect to control BGLs^42,43^. Therefore, insulin, chosen as a model drug, was incorporated into the charged nanovesicles for transdermal administration. Due to the various advantages of iontophoresis in delivering charged molecules, electropositive nanovesicles are expected to synergize in order to regulate the BGL when combined with the MAs and iontophoresis. The nanovesicles effectively entrap insulin with a high efficiency of ~88%. The insulin concentration in nanovesicles was 29.3 IU/mL. The insulin-loaded nanovesicles possess a spherical shape with a defined edge. The size distribution of the insulin-loaded nanovesicles was analyzed with dynamic light scattering (Fig. 2h, insert). The average diameter, zeta potential, and polydispersity index of the nanovesicles are ~230.3 nm, +10.28 mV, and 0.25, respectively. These electropositive insulin-loaded nanovesicles were loaded in the medical sponge of the IMAP for the treatment of type 1 diabetes.

### Mechanical performance

**Fig. 3:**
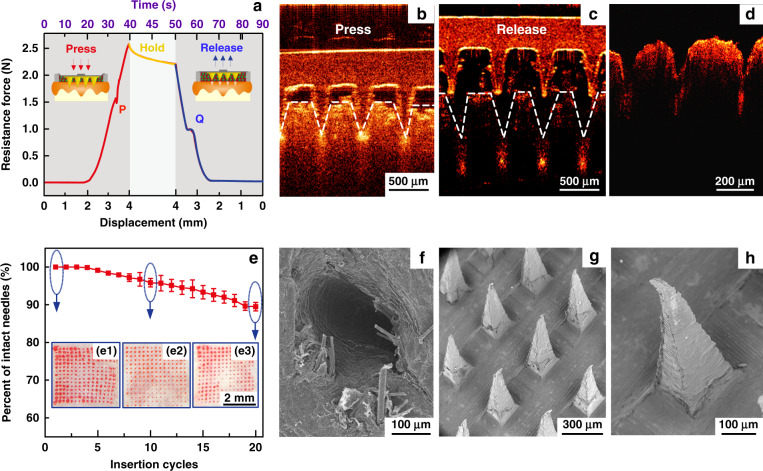
Mechanical performance of the IMAP. **a** Mechanical curves of the IMAP during the P&R test. **b** OCT images of skin penetration by the MA during the press stage, **c** solid microneedles retract from the skin to the medical sponge during the release stage, and **d** poked skin after the ‘P&R’ test. **e** The mechanical stability of the MA assessed by the insertion ratio of the intact poked microholes to the microneedle number, and pictures of skin samples treated with the IMAP at (e1) the 1st, (e2) the 10th and (e3) the 20th cycle of the ‘P&R’ action. **f** SEM image of the microhole in the rabbit skin poked by the IMAP after the 20th cycle, and **g**–**h** SEM images of the MA of the IMAP after the 20th cycle.

The resistance force of the IMAP under the ‘P&R’ test is shown in Fig. 3a, and the locally magnified curves of point ‘P’ and point ‘Q’ are shown in Fig. S7. In the “Press stage”, the resistance force gradually increases with the compression loads on the IMAP. Once the resistance force reaches the rupture limit, the MA pierces into the skin, corresponding to a drop at point ‘P’. The critical penetration force and energy are 1.6 N and 7.93 mJ, respectively. Thus, the IMAP can readily penetrate into skin using a thumb of an adult whose average compression force could reach ~20 N^44^. The microneedles penetrate deeply and create a microhole array in the skin with continuously increasing compression. The optical coherence tomography (OCT) image of skin penetration by the MA of the IMAP corresponds to the “Press stage” of the above force and displacement curves, as presented in Fig. 3b. The MA has already broken the SC and has embedded in the penetrated skin rather than staying in the sponge reservoir when the IMAP was pressed. The result visually demonstrates that the MA can pass through the medical sponge and pierce into the skin, creating microholes through the SC layer. In the “release stage”, the measured force gradually decreases with the release of compression on the IMAP. Once the MAs are detached from the skin, the friction decreases to 0, corresponding to an increase at point ‘Q’, as shown in Fig. 3a. Finally, the MA completely retracts into the medical sponge, with the corresponding microholes remaining in the skin, as shown in Fig. 3c. After the ‘P&R’ test, the microholes can be clearly observed by using the OCT image of the poked skin, as shown in Fig. 3d. The average depth and base width of the poked microholes are ~200 and 100 μm, respectively.

The poked microholes created by the IMAP will gradually self-close due to the recovery ability of the skin. Thus, repeated ‘P&R’ actions can be executed to reopen the pathway for drug diffusion and iontophoresis. It is necessary to investigate the mechanical stability of the IMAP, which can be evaluated by the insertion ratio. The poked skin samples after the 1st, 10th and 20th cycles of ‘P&R’ action were observed using SEM, as shown in Fig. 3e1–e3. A 100% insertion ratio was observed in the initial four cycles, showing an excellent strength of the MA. The insertion ratio gradually decreased to 96% at the 10th cycle and 89% at the 20th cycle, indicating that MA still had enough mechanical strength for skin penetration after 20 cycles of ‘P&R’ actions. The microhole poked by the IMAP after the 20th cycle is shown in Fig. 3f, verifying successful skin penetration using the IMAP. The SEM image of the MA after the 20th cycle is shown in Fig. 3g. A few microneedles were bent slightly but still intact, which might be the reason for the failure of microhole insertion. The experiment also demonstrated that the MA possesses satisfactory toughness, which means that the microneedles remain intact after multiple cycles of penetration. Therefore, the MA in the IMAP has excellent mechanical stability for repeated ‘P&R’ actions to reopen the closing microholes. Above all, the penetration force of the MA is far less than the average compression force of an adult using a thumb; thus, the IMAP can easily penetrate into the skin using finger compression. At this moment, the MA passes through the sponge reservoir and embeds in the punctured skin. Once the compression is released, the MA is detached from the skin and retracts into the sponge reservoir, which forms a complete ‘P & R’ action. The integrity without fracture of the MA after twenty ‘P & R’ actions exhibits excellent toughness to realize repeated puncture. Furthermore, the MA was prepared from PMMA, which has excellent mechanical resistance and satisfactory biocompatibility, which also reduces the risk of skin irritation.

### In vitro transdermal insulin delivery performance

**Fig. 4:**
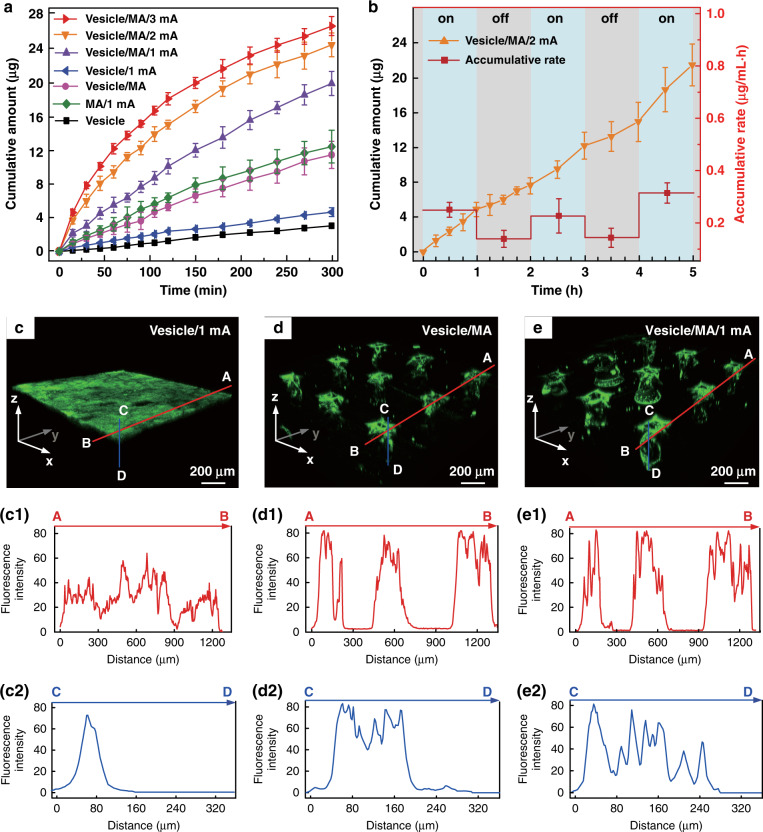
In vitro transdermal insulin delivery performance of the IMAP. **a** The cumulative permeation amount of FITC-insulin using various IMAP groups including the vesicle group, vesicle/MA group, insulin/MA/1mA group, vesicle/1mA group, vesicle/MA/1mA group, vesicle/MA/2mA group and vesicle/MA/3mA group. **b** In vitro cumulative permeation amount and the rate of FITC-insulin in the vesicle/MA/2mA groups alternated with power-on (blue area) and power-off (gray area). **c** The 3D confocal reconstruction images of rabbit skins treated with the vesicle/1mA group, and its fluorescence distribution along the marked red line (c1) and blue line (c2). **d** 3D confocal reconstruction images of rabbit skins treated with the vesicles/MA, and the distribution of the fluorescence intensity along with the red (d1) and blue lines (d2). **e** The 3D confocal reconstruction images of rabbit skins treated with the vesicle/MA/1mA group, and its fluorescence distribution along the red line (e1) and blue line (e2). The red line indicates the point ‘A’ to point ‘B’ on the *Y* axes of the reconstruction images, while the blue line indicates the point ‘C’ to point ‘D’ on the *Z* axes.

In addition to excellent mechanical performance, a qualified transdermal drug delivery device also requires an effective and controlled drug diffusion capability. Thus, in vitro permeation experiments were conducted by using Franz diffusion cells, as shown in Fig. S5. The IMAPs under different usage conditions were divided into seven groups (vesicle group, vesicle/MA group, insulin/MA/1 mA group, vesicle/1 mA group, vesicle/MA/1 mA group, vesicle/MA/2 mA group and vesicle/MA/3 mA group, as described in the “Materials and methods” section) to be employed as the upper donor chamber. The details of each group are listed in Table 1 and illustrated in Fig. S6. The in vitro fluorescein isothiocyanate (FITC)-insulin cumulative permeation amount of each group was recorded in the same chart, as presented in Fig. 4a. The percentage of the total cumulative amount of FITC-insulin released from the reservoirs within 5 h is shown in Fig. S8. The cumulative amount of FITC-insulin increases with the treatment period. After 5 h of exposure, a significant difference in cumulative permeation was clearly observed among these groups. The effect of the MA of the IMAP on drug permeation was first investigated. The cumulative permeations of FITC-insulin for groups without MA administration, including the vesicle group and vesicle/1 mA group, were 3.10 ± 0.02 and 4.85 ± 0.57 µg, respectively. Conversely, the cumulative amounts for the groups with MA compression, such as the vesicles/MA group and vesicles/MA/1 mA group, were 11.36 ± 1.52 and 21.41 ± 1.33 µg, respectively. Thus, it is obvious that the cumulative permeation amount of FITC-insulin in the vesicle/MA group is increased by 3.7 times compared with the vesicle group. The cumulative permeation amount of FITC-insulin in the vesicle/MA/1 mA group was ~4.4-fold higher than that in the vesicle/1 mA group. This result indicates that the MA can effectively promote the passive diffusion of insulin-loaded nanovesicles attributed to the skin microholes created by microneedles. The microholes greatly overcame the skin barrier and were beneficial for a significant increase in the permeation rates. This passive permeation profile of the insulin-loaded nanovesicles through rabbit skin was in accordance with Fick’s law. The cumulative amount of FITC-insulin in the vesicle/1 mA group was only increased 1.5 times compared with the vesicle group, indicating that the MA possessed a more powerful capability to improve permeation performance than iontophoresis.

**Table 1:** IMAP groups under different usage conditions for in vitro insulin delivery.

Group name	Loading drug	MA Penetration	Iontophoresis
Vesicle	FITC-insulin loaded vesicles	–	–
Vesicle/MA	FITC-insulin loaded vesicles	10 N compression	–
Insulin/MA/1 mA	Free FITC-insulin	10 N compression	1 mA
Vesicle/1 mA	FITC-insulin loaded vesicles	–	1 mA
Vesicle/MA/1 mA	FITC-insulin loaded vesicles	10 N compression	1 mA
Vesicle/MA/2 mA	FITC-insulin loaded vesicles	10 N compression	2 mA
Vesicle/MA/3 mA	FITC-insulin loaded vesicles	10 N compression	3 mA

The permeation rate with the combination of the MA and iontophoresis was also investigated. The cumulative permeation amounts of FITC-insulin were 11.36 ± 1.52, 21.41 ± 1.33, 23.91 ± 1.33 and 25.48 ± 1.14 µg for the vesicle/MA group, vesicle/MA/1 mA group, vesicle/MA/2 mA group, and vesicle/MA/3 mA group, respectively. The permeation rate of the vesicle/MA/3 mA group was the highest, which was ~2.24 times higher than that of the vesicle/MA group. The significant increase is attributed to the positive effects of electroosmosis and electrostatic interactions^40^. The cumulative permeation amount almost linearly increases with the intensity of the applied electric field. Thus, the synergy between the MA and iontophoresis is beneficial for promoting and controlling drug delivery. The nanovesicles in the IMAP also contribute to the drug permeation of insulin; the cumulative amount of FITC-insulin in the vesicle/MA/1 mA group was 1.74-fold that in the insulin/MA/1 mA group (12.29 ± 0.19 µg). The charged nanovesicles are favorable for transporting the encapsulated insulin across the SC under the electrophoresis force of iontophoresis. The electrostatic attraction between the skin and electropositive nanovesicles also contributes to improving the permeability coefficient of insulin molecules and partition coefficient^37^. On the other hand, as drug carriers, nanovesicles are advantageous for potentially sustained drug release, reducing toxicity and improving long-term stability^27^.

Therefore, the integrated effects of the MA and iontophoresis can achieve a remarkable effect in the transdermal delivery of insulin-loaded nanovesicles.

The in vitro cumulative permeation amount and rate of FITC-insulin in the vesicle/MA/2 mA group alternate with the ‘power on-off’ cycles, as shown in Fig. 4b. The accumulative permeation rate of FITC-insulin fluctuates well with the ‘power on-off’ cycle (with and without application of 2 mA current) in a step-like manner. Clearly, the accumulative permeation rates at ‘power-on’ are 0.26, 0.24, and 0.31 µg/mL h, respectively, which are almost twice those at ‘power-off’. This result further demonstrates the enhancement of the transdermal insulin permeation rate driven by iontophoresis and the feasibility of on-demand drug administration in an active and easy manner.

The 3D confocal reconstruction images of rabbit skins treated with the vesicles/1 mA group, vesicles/MA group, and vesicles/MA/1 mA group for one-hour administration are shown in Fig. 4c–e, respectively. The different cross sections of the 3D confocal reconstruction images can be seen in Fig. S9. In addition, the total fluorescence intensity and the fluorescence intensity along the marked red and blue lines of the above images are also shown in Fig. S10, Fig. 4c1–e1 and Fig. 4c2–e3, respectively. Without skin penetration by the MA, most FITC-insulin driven by iontophoresis remains on the skin surface owing to the good barrier of the intact SC layer, as shown in Fig. 4c–c1. The insulin permeation depth is only ~90 µm, as shown in Fig. 4c2. However, insulin can effectively diffuse through the microholes in the skin poked by the MA, enhancing permeation at a greater depth of ~210 µm, even in the absence of iontophoresis, as seen in Fig. 4d–d2. The fluorescence intensity is the highest where there are microholes, which means that almost all insulin can diffuse to the deeper layers of skin because of these microholes are created by the MA. Furthermore, the synergistic therapy of the MA and iontophoresis exhibits the deepest transdermal permeation depth of ~280 µm, as shown in Fig. 4e–e2. Iontophoresis, forming a potential difference as a driving force, further improves the permeation of insulin-loaded nanovesicles through poked skin. Above all, the IMAP integrated with MA, iontophoresis and charged nanovesicles can achieve a synergistic and remarkable enhancement in transdermal delivery.

### In vivo studies using diabetic rats

**Fig. 5:**
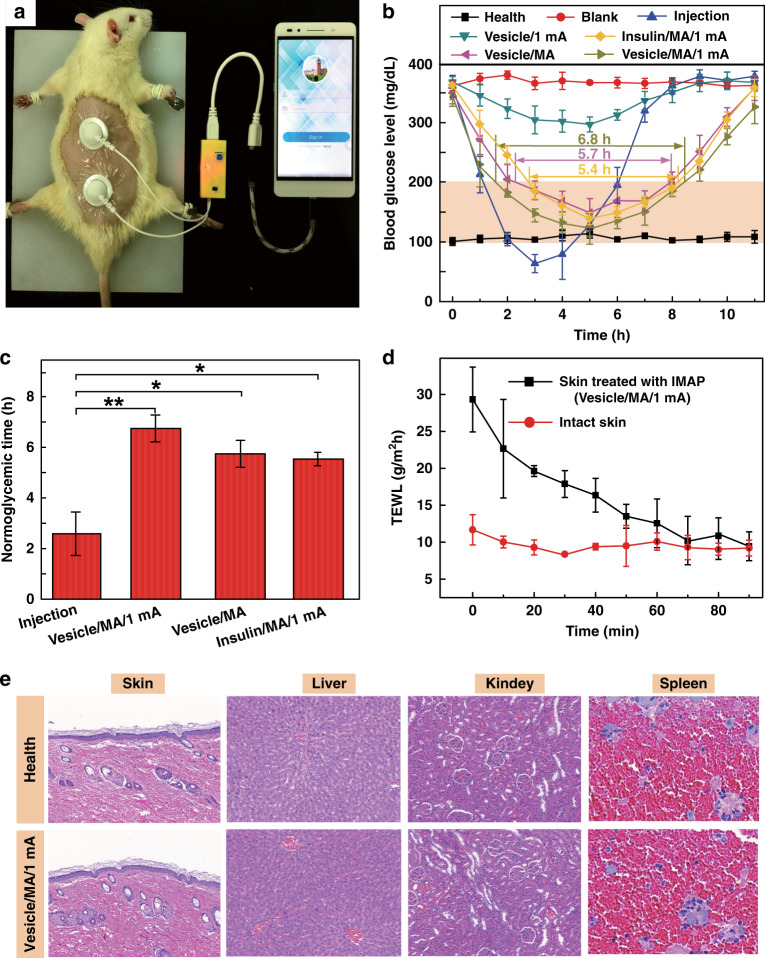
In vivo studies of the IMAP for type 1 diabetes treatment. **a** Image of the IMAP and its smartphone-powered iontophoresis-driven device for the treatment of a type 1 diabetic SD rat. The rat dorsum and relevant skin (the area was unhaired beforehand) were transcutaneously treated with a pair of the IMAPs connected with an iontophoresis-driven circuit. **b** BGLs of diabetic rats of each group administered different transdermal insulin delivery approaches, including the health group, blank group, injection group, vesicle/1mA group, insulin/MA/1mA group, vesicle/MA group, and vesicle/MA/1mA group. **c** Histogram of the mean normoglycemic times of the injection group, vesicle/MA/1mA group, vesicle/MA group, and insulin/MA/1mA group. **d** TEWL curves of poked skin in the vesicle/MA/1mA group and intact skin in the health group. **e** Hematoxylin and eosin (H&E) staining of the main organs in the vesicle/MA/1mA group and health group.

Transdermal insulin administration has been hailed as an alternative for diabetes treatment owing to various features, such as painless self-administration and good compliance^12,45^. To demonstrate the advantage of IMAP drug administration strategies, in vivo transdermal insulin therapy using IMAPs was studied in streptozotocin (STZ)-induced diabetic rats (Fig. 5a). The self-developed smartphone-powered drug delivery system was used for the treatment of type 1 diabetic rats owing to the convenient use of the IMAP and the portability of the miniature iontophoresis-driven device.

The BGLs of the rats treated in different groups were investigated (Fig. 5b), and the corresponding normoglycemic times were also calculated (Fig. 5c). Diabetic rats whose BGL is in the range of 100–200 mg/dL are regarded as in the normoglycemic state^22,46^, and the effective period under this state is defined as the normoglycemic time. The details of each group are described in the Materials and Methods section and listed in Table 2. The BGLs of the healthy rats and diabetic rats remained steady at 108.6 ± 10.5 mg/dL and 370.2 ± 14.3 mg/dL, respectively. The BGL of the subcutaneous injection group decreased sharply to 63.6 ± 15.1 mg/dL with hypoglycemia^12^ and then rapidly rebounded to the initial state. The mean normoglycemic time of the injection group was ~2.2 h. Therefore, the subcutaneous injection of insulin has a strong hypoglycemic effect but may induce a risk of hypoglycemia. In addition, subcutaneous injection is usually painful and invasive, while the MA of the IMAP is fairly gentle for patients.

**Table 2:** Rat groups for in vivo transdermal insulin delivery.

Group name	Rats	Loading drug	MA Penetration	Iontophoresis
Health group	Healthy rats	–	–	–
Blank group	Diabetic rats	–	–	–
Injection group	Diabetic rats	Insulin-loaded vesicles	–	–
Vesicle/1 mA group	Diabetic rats	Insulin-loaded vesicles	–	1 mA
Insulin/MA/1 mA group	Diabetic rats	Free insulin	10 N compression	1 mA
Vesicle/MA group	Diabetic rats	Insulin-loaded vesicles	10 N compression	–
Vesicle/MA/1 mA group	Diabetic rats	Insulin-loaded vesicles	10 N compression	1 mA

Correspondingly, the IMAP combined with the MA, iontophoresis and nanovesicles also exhibited hypoglycemic effects (Fig. 5b, c). Iontophoresis coupled with electropositive nanovesicles can slightly lower the BGL of diabetic rats. The BGL of diabetic rats in the vesicle/1 mA groups slightly decreased within the initial 5 h at a minimum BGL of 303 ± 7.8 mg/dL. This result reveals that even iontophoresis does not promote macromolecular insulin (~5.8 kDa) permeation through intact skin^47^. MAs are more beneficial for the transdermal delivery of nanovesicles than for iontophoresis. The BGL of diabetic rats in the vesicle/MA group gradually decreased to a minimum level of 156.9 ± 15.4 mg/dL and was maintained at the normoglycemic state for 5.7 ± 0.5 h. The MA provided a large number of microholes in the skin, eliminating the effect of the SC barrier and increasing the permeability coefficient. The synergetic application of the MA, iontophoresis, and nanovesicles exhibits the best hypoglycemic effect. The BGL of diabetic rats in the vesicle/MA/1 mA group rapidly decreased to a minimum level of 121.8 ± 25.5 mg/dL and was maintained at the normoglycemic state for 6.7 ± 0.5 h. The initial decrease in the speed of the BGL in the vesicle/MA/1 mA group was comparable to that in the injection group. This finding implies that the amalgamation of the MA with iontophoresis and nanovesicles can decrease the time lag, thereby accelerating therapy via transdermal delivery. The normoglycemic time of diabetic rats in the vesicle/MA/1 mA group was ~3.1-fold and 1.2-fold that in the injection group and vesicle/MA group, respectively. A more perspicuous statistical graph of the vesicle/MA/1 mA group, injection group and vesicle/MA group and their detailed BGLs measured by a blood glucose meter are shown in Fig. S11–S12. The normoglycemic time in the vesicle/MA/1 mA group was ~1.2 times that in the vesicle/MA group, demonstrating the excellent capacity of iontophoresis to enhance transdermal insulin penetration. This positive promotion could be explained by the electrical force from iontophoresis. As the driving force, it contributes to the active transport of nanovesicles through microholes and an enhancement of the diffusion coefficient^37^.

MA-induced microholes create many pathways on skin for nanovesicle permeation. Nanovesicles, as carriers of insulin, are effectively delivered along the pathway under the synergistic effect of passive diffusion and active iontophoresis. The normoglycemic time of diabetic rats in the vesicle/MA/1 mA group was ~1.26 times that in the insulin/MA/1 mA group (5.5 h ± 0.3 h), demonstrating that nanovesicles contribute to the increase in the iontophoresis coefficient^37^. Above all, the IMAP combined with MA, iontophoresis and nanovesicles provides a valuable alternative for the effective and long-lasting transdermal delivery of macromolecular drug solutions.

The safety of the IMAP was evaluated for possible clinical application, as shown in Fig. 5d, e. Figure 5d presents the transepidermal water loss (TEWL) curves of poked skin in the vesicle/MA/1 mA group and intact skin in the health group. The TEWL of poked skin decreases to the level of intact skin after the removal of the IMAP, indicating that the impaired skin by the MA can rapidly recover within 90 min. The closure of MA-induced microholes may restrain the pervasion of liquid macromolecular drugs. Thus, the ‘P&R’ actions should be repeated every 1 h to recreate the microholes for continuous delivery. However, it indirectly demonstrates that IMAP is minimally invasive for users. The possible effect of skin penetration by the MA and the driven current of iontophoresis on the safety of diabetic rats was investigated. Slices of the skin, liver, kidney and spleen of diabetic rats in the vesicle/MA/1 mA group and health group were observed, as shown in Fig. 5e. In both the vesicle/MA/1 mA group and the health group, no significant density increase in the infiltrating inflammatory cells in the skins, no abnormal characteristics, including the foamy appearance of hepatic cells and disintegration of the hepatic cords in the livers, no extensive nephron damage or glomeruli collapse in the kidneys, and no obvious damage in the spleens were observed^48^. These in vivo studies reveal encouraging findings that the IMAP is safe for receptors, which may significantly improve compliance and curative effects.

## Conclusions

In summary, we developed a novel IMAP and its matching smartphone-powered iontophoresis-driven device for the efficient and controlled transdermal delivery of insulin. The IMAP is a combination of an MA, iontophoresis and charged nanovesicles, which executes the drug administration strategy “Penetration, Diffusion and Iontophoresis”. The MA of the IMAP can reopen the self-healing microholes for long-lasting diffusion, and iontophoresis enables further enhancement of controlled insulin delivery. In vitro experiments demonstrated that the accumulative rate of insulin could be increased by iontophoresis and that the total amount could be significantly regulated by applying different current intensities, which contributes to efficient and on-demand insulin administration. In vivo studies showed that IMAP-coupled iontophoresis and nanovesicles were fairly effective in BGL control with the reliable avoidance of hypoglycemia side effects. Furthermore, the utilization of the IMAP under mild current intensity is safe enough without raising safety concerns. Above all, the IMAP can shake off the traditional designs of the MA and injection needles and offer a more powerful tool with improved efficiency for macromolecular drug delivery in a controllable manner.

## Materials and methods

### Ethics statement

The details are presented in the Supporting Information.

### Chemicals and animals

Soybean lecithin, propylene glycol (Tianjin Chemical Reagent Co., Ltd., China), and cetyltrimethyl ammonium bromide (Shanghai Aobo Chemical Reagent Co., Ltd., China) were purchased for the preparation of the nanovesicles. MA female molds (Micropoint Technologies Pte Ltd., Singapore) were purchased for the fabrication of the MA using a micromolding method. FITC, STZ and bovine pancreas insulin were purchased from Sigma-Aldrich (USA).

A New Zealand rabbit (male, 2–3 months, 3.0 kg) was offered by the Xinhua Experimental Animal Farm (Huadu district, Guangzhou). The rabbit was euthanized for hair removal. The rabbit skin was cut to an appropriate size. SD rats (male, 200 ± 30 g) were provided and quarantined from the Experimental Animal Center of Sun Yat-Sen University.

### Assembly of the IMAP

The PMMA MAs were fabricated by a typical micromolding method, as presented in Fig. S2. The MA of the IMAP was characterized using SEM (Quanta 400 F, Oxford, Holland). A medical tape, a conductive film with an electrode, an MA, a gasket, and a medical sponge loaded drugs were assembled as an IMAP, as shown in Fig. 1a.

A miniature iontophoresis-driven printed circuit board (PCB, 35 mm × 15 mm ×2 mm) was developed to output an adjustable constant current for the IMAP, as shown in Fig. S3a, b. The insulation shell (40 mm× 20 mm ×15 mm) of iontophoresis-driven PCB was fabricated by a 3D-printer (Sindon, 3DWOX, Korea) for electrical safety, as shown in Fig. S3a.

### Preparation and characterization of the insulin-loaded nanovesicles

The insulin stock solution was first prepared by dissolving insulin (27 IU/mg) in phosphate-buffered saline (PBS). Soybean lecithin and propylene glycol at a mass ratio of 1:2 was uniformly mixed, poured into insulin stock solution, homogenized using the ultrasound method for 20 min, and subsequently added to 0.4% cetyltrimethylammonium bromide (CTAB). Finally, insulin-loaded nanovesicles were constructed. FITC-labeled insulin-loaded nanovesicles were also prepared by performing similar procedures. The nanovesicles were observed using TEM (JEOL-2100F, Japan) and were measured by photon correlation spectroscopy (Nano ZS90, Malvern Instruments, U.K.).

### Mechanical tests

A customized test equipment was designed for mechanical tests, as illustrated in Fig. S4. A 150 μL sulforhodamine B solution (0.4 wt%) was filled in the porous sponge. The skin penetration process of the IMAP was performed by mimicking the “P&R” action on rabbit skin. The IMAP was gradually pressed at 4 mm, held for 10 s, and subsequently released the compression. Both P&R velocities are 0.1 mm/s. The whole process was simultaneously recorded and observed using OCT (HSO-2000, TEK SQRAY, China).

The mechanical stability of the IMAP was studied by repeating ‘P&R’ actions for 20 cycles on rabbit skin at different locations. The residual sulforhodamine B solution was washed off after removal of the IMAP. The red dots remaining on the skin indicate that the microholes were successfully poked by microneedles. The mechanical stability was evaluated by the insertion ratio of the microholes poked by IMAP and the microneedle number. The poked holes in the skin and the MA of used IMAP were observed using a microscope (BX51 M, Olympus, Japan) and SEM.

### In vitro transdermal insulin delivery

The receptor chamber of Franz diffusion cells (TP-3A, Albert Tech., China) was fully filled with PBS and stirred constantly at 37 ± 1 °C. Drug-loaded IMAP was employed as the upper donor chamber. As shown in Fig. S5, the IMAP taped on the rabbit skin and Ag/AgCl electrode inserted in the PBS of the receptor chamber were employed as the anode and cathode of iontophoresis, respectively. IMAPs with 150 μL FITC-insulin loaded vesicles, 10 N compression once per hour, and 1–3 mA iontophoresis were proposed. Thus, IMAPs under different usage conditions were divided into seven groups. (1) Vesicle group: IMAP filled with FITC-insulin loaded vesicles without application of compression and iontophoresis, (2) insulin/MA/1 mA group: IMAP filled with free insulin under 10 N compression and 1 mA iontophoresis, (3) vesicle/MA group: IMAP filled with FITC-insulin loaded vesicles under 10 N compression without iontophoresis, (4) vesicle/1 mA group: IMAP filled with FITC-insulin loaded vesicles under 1 mA iontophoresis, (5) vesicle/MA/1 mA group: IMAP filled with FITC-insulin loaded vesicles under 10 N compression and 1 mA iontophoresis, (6) vesicle/MA/2 mA group: IMAP filled with FITC-insulin loaded vesicles under 10 N compression and 2 mA iontophoresis, and (7) vesicle/MA/3 mA group: IMAP filled with FITC-insulin loaded vesicles under 10 N compression and 3 mA iontophoresis.

The cumulative amount of FITC-insulin was determined by fluorescence spectroscopy. In addition, the 2 mA iontophoresis current of the vesicle/MA/2 mA group was turned ‘on-off’ every hour for 5 cycles for further investigation. The FITC-insulin permeation processes through the rabbit skin after treatment by the vesicle/MA group, vesicle/1 mA group, and vesicle/MA/1 mA group for one hour were observed and reconstructed by a confocal laser scanning microscope (CLSM, Zeiss 710, Germany).

### In vivo studies for diabetic rats

Sprague-Dawley (SD) rats were fasted for 18 h and injected with STZ solution (10 mg/mL, 55 mg/kg) to induce type-1 diabetes. Their BGLs were detected for at least 3 d. The rats with a BGL that was kept steady over 300 mg/dL were induced successfully. A pair of IMAPs filled with insulin and physiological saline were used as the anode and cathode for the iontophoresis process. The center-to-center distance of the two IMAPs was set to ~35 mm. The rats were divided into eight groups to study the 5 IU insulin administrations as following: (1) health group: healthy rats; (2) blank group: diabetic rats with no treatment; (3) Injection group: diabetic rats were subcutaneously injected of 5 IU insulin loaded nanovesicles; (4) vesicle/1 mA group: diabetic rats treated with the IMAP (loading drug: 5 IU insulin loaded nanovesicles; MA penetration: no compression; iontophoresis-driven current: 1 mA); (5) insulin/MA/1 mA group: diabetic rats treated with the IMAP (loading drug: 5 IU free insulin solution; MA penetration: 10 N compression once per hour; iontophoresis-driven current: 1 mA); (6) vesicles/MA group: diabetic rats treated with the IMAP (loading drug: 5 IU insulin loaded nanovesicles; MA penetration: 10 N compression once per hour; iontophoresis-driven current: 0 mA); and (7) vesicles/MA/1 mA group: diabetic rats treated with the IMAP (loading drug: 5 IU insulin loaded nanovesicles; MA penetration: 10 N compression once per hour; iontophoresis-driven current: 1 mA). Blood was extracted from the tail vein and detected by a blood glucose meter (Sinocare Inc., China).

### TEWL and organ slices

The back hair of SD rats was shaved one day in advance. A drug-loaded IMAP was applied and performed a ‘P&R’ action and powered with 1 mA iontophoresis. The TEWL of the punctured skin was measured by a Vapometer (Delfin Technologies, Finland).

The skins, livers, kidneys and spleens of the diabetic rats in the vesicle/MA/1 mA group and health group after insulin treatment for 12 h were dissected and H&E stained. These organ slices were observed via an optical microscope.

### Statistical analysis

The data were calculated and expressed as the mean ± standard deviation. The differences between the two compared groups were determined by Student’s *t*-test. *p* values < 0.05 indicated that the results were considered statistically significant. *p values < 0.05, **p values < 0.01.

## Supplementary information

Supplementary Video of the smartphone-based drug delivery system

Supplementary Information
