# Supplementary material for: Smartphone-powered iontophoresis-microneedle array patch for controlled transdermal delivery
Source: Microsyst Nanoeng. 2020 Dec 28;6:112. doi: 10.1038/s41378-020-00224-z (PMC8433361; doi:10.1038/s41378-020-00224-z)
Supplement: Supplementary file 2 — Supplementary Information [file 41378_2020_224_MOESM2_ESM.pdf]

Supporting Information for

Smartphone-powered Iontophoresis-Microneedle Array Patch for Controlled Transdermal Delivery

Jingbo Yang [a], Yanjun Li [a], Rui Ye [a], Ying Zheng [a], Xiangling Li [a,b] ,Yuzhen Chen [a],

Xi Xie [b, *], Lelun Jiang [a,*]

[a]Guangdong Provincial Key Laboratory of Sensor Technology and Biomedical Instrument, School of Biomedical Engineering, Sun Yat-Sen University, Guangzhou 510275, China;

[b]State Key Laboratory of Optoelectronic Materials and Technologies, School of Electronics and Information Technology, Sun Yat-sen University, Guangzhou 510275, China.

*Authors for correspondence: jianglel@mail.sysu.edu.cn; xiexi27@mail.sysu.edu.cn

1. Drug delivery mechanism of “Penetration, Diffusion and Iontophoresis”

The transdermal drug administration strategy of IMAP is “Penetration, Diffusion and Iontophoresis”, including drug diffusion across intact skin, drug diffusion across poked skin, drug diffusion and iontophoresis across intact skin, and drug diffusion and iontophoresis across poked skin. Briefly, since the IMAP is pressed, MA soaked in the porous reservoir will penetrate through the sponge and disrupt the SC, creating transient aqueous micro-holes. As the compression on IMAP is released, the MA will retract the sponge again. MA-induced micro-holes are directly exposed to the drug solution stored in the sponge. According to Fick’s law, the drug solution will diffuse into the skin through micro-holes. Then, a mild electric current is conducted between a pair of electrodes, to drive charged drug molecules through micro-holes into systemic circulation by the predominant driving forces of electromigration and electroosmosis. One of the key advantages of this design is that the users can repeat the touch-actuated ‘press and release’ processes to reopen the micro-holes for a new round drug administration, resulting in a long-lasting positive diffusion and active iontophoresis.

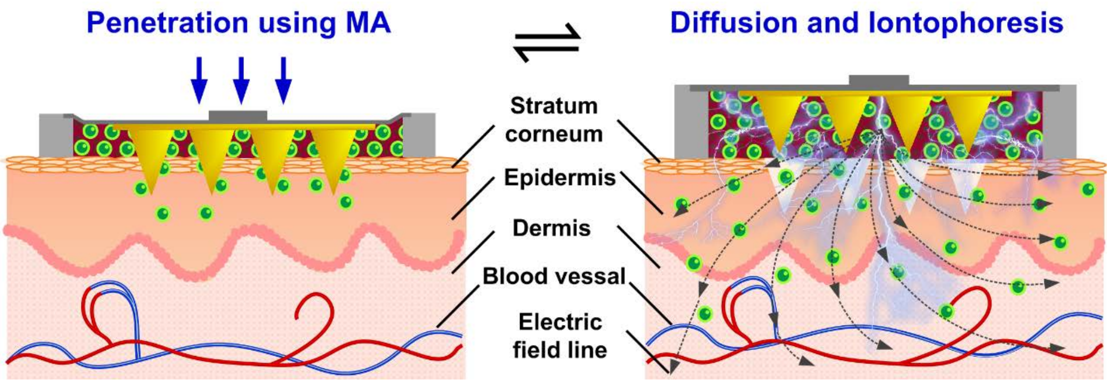

Figure S1 Schematic representation of the drug delivery mechanism of IMAP: "Penetration, Diffusion and Iontophoresis". Since the IMAP is pressed, the micro-holes were created in the skin. Once compression is removed, the MA will retract in reservoir again. The drug solution will passively diffuse into the skin through micro-holes. And active iontophoresis will drive charged therapeutic molecules into systemic circulation when electric current is conducted.

2. Fabrication of PMMA solid MA by micromolding technique

Methyl methacrylate (MMA) and benzoyl peroxide (BPO) were purchased from Zhiyuan Chemical Reagent Co., Ltd, China. Polydimethylsiloxane (PDMS, Sylgard 184) was bought from Dow Corning, England. MA female molds (Micropoint Technologies Pte Ltd, Singapore) were purchased for the fabrication of solid MA. Micromolding technique, suitable for mass production, was proposed to fabricate solid MA. The detailed fabrication process is presented in Figure S2. MMA was uniformly mixed with BPO at a weight ratio of 71.4: 1 to prepare the pre-polymerized PMMA at a temperature of 80 °C for 40 min. The pre-polymerized PMMA was casted in the female PDMS mold under a vacuum of approximately 2000 Pa for a night. The PMMA MA was solidified in the PDMS mold and peeled off from PDMS mold. Finally, the PMMA solid MA was fabricated.

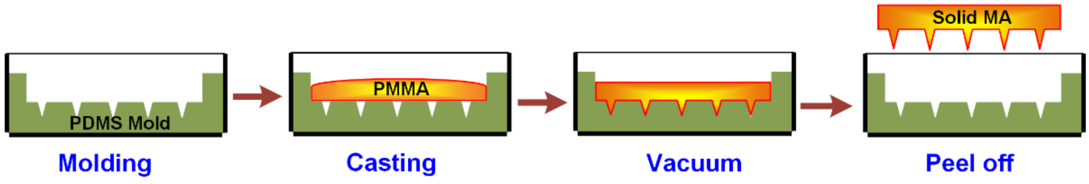

Figure S2 Fabrication process of PMMA solid MA using micro-molding technique

3. The iontophoresis-driven circuit

Figure S3a shows the miniature iontophoresis-driven device for transdermal drug delivery with a size of 40 mm × 20 mm × 15 mm. The PCB of iontophoresis-driven circuit (Figure S3b) was encapsulated with a 3D printed shell. The iontophoresis-driven circuit mainly consists of two modules: input voltage stabilization and output constant current (Figure S3c). The iontophoresis-driven circuit can be powered through the smartphone charging port or USB charger. The power is rectified and the input voltage is stabilized using a low dropout voltage regulator (AMS1117, Zhiquan Electronics Fittings Factory, China) and two capacitor filters. The obtained stable voltage V_{CC} is transferred into an output constant current. The output constant current I_{C2} is achieved using circuit of Wilson current source, which mainly contains three identical PNP-triodes (2N 3906B331), as shown in Figure S3d. Triode T_0 and T_1

are connected in a mirror symmetry, thus I_{B0}=I_{B1}= I_B and I_{C0}=I_{C1}= I_C. The emitter of triode T_2 is connected with the base and collector of T1 in series. There is a large equivalent resistance *R* between the emitter and collector, so the output current can be well stabilized. The detailed The derivation process of output constant current I_{C2} is

The current equation at point Q is

$$I_{E2} = I_{C1} + 2I_B = I_{C1} + \frac{2I_{C1}}{\beta} = I_{C1}\frac{2+\beta}{\beta} \tag{1}$$

in which β is the triode gain. The I_{E2} also is equal to

$$I_{E2} = I_{B2} + I_{C2} = \frac{I_{C2}}{\beta} + I_{C2} = \frac{\beta+1}{\beta}I_{C2} \tag{2}$$

According to Eq. (1) and Eq. (2), we can get

$$I_{C1} = \frac{\beta}{\beta+2}\cdot I_{E2} = \frac{\beta}{\beta+2}\cdot\frac{\beta+1}{\beta}I_{C2} = \frac{\beta+1}{\beta+2}\cdot I_{C2} \tag{3}$$

The current equation at point P is

$$I_R = I_{B2} + I_{C0} = \frac{I_{C2}}{\beta} + \frac{\beta+1}{\beta+2}\cdot I_{C2} = \frac{\beta^2+2\beta+2}{\beta^2+2\beta}\cdot I_{C2} \tag{4}$$

When $\beta > 10$

$$I_{C2} = \left(1 - \frac{2}{\beta^2+2\beta+2}\right) I_R \approx I_R \tag{5}$$

So, the output current I_{C2} is approximately equal to I_R, which is independent of the external resistance.

Furthermore, the output current can be adjusted by changing the internal load through a switch (SW-SPDT). Finally, the iontophoresis-driven circuit can output constant different currents of 1 mA, 2 mA and 3 mA by adjustment of the equivalent resistance *R*.

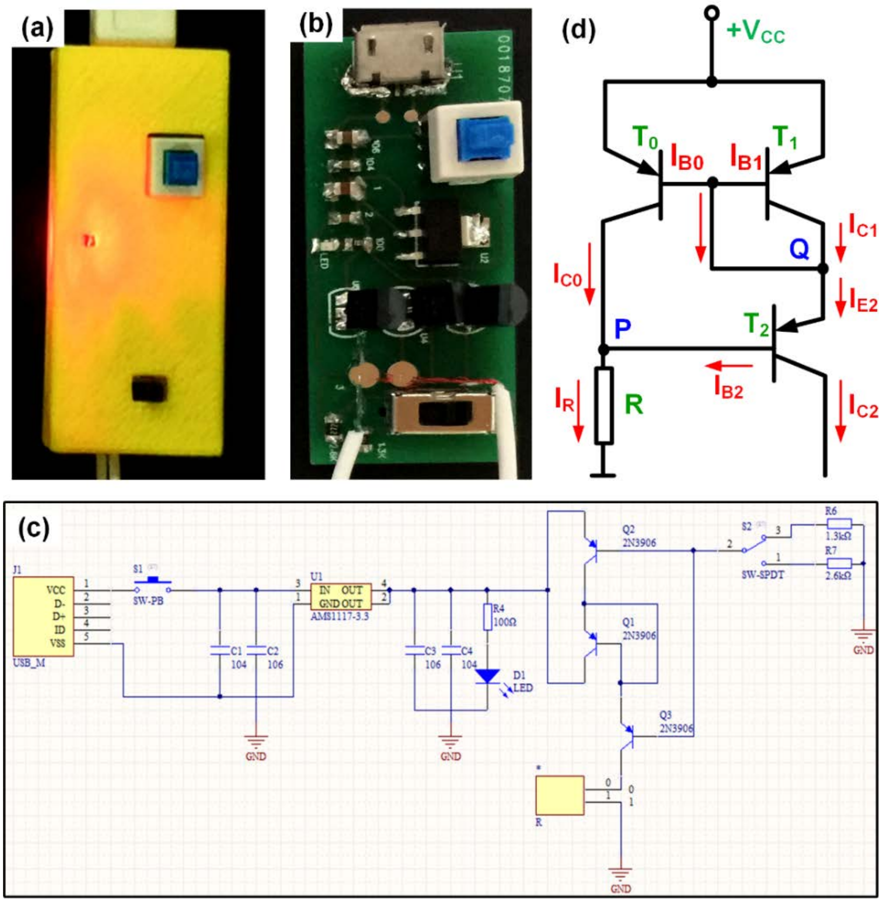

Figure S3 (a) The photo of miniature iontophoresis-driven device for transdermal drug delivery; (b) The PCB photo of iontophoresis-driven circuit. (c) The detailed schematic diagram of iontophoresis-driven circuit. (d) The detailed schematic diagram of constant current output.

4. Mechanical loading setup

A custom-made mechanical loading setup was developed for the investigation of mechanical performance of IMAP, as shown in Figure S4. A force sensor (Nano 17 Titanium, ATI Industrial Automation, USA) is assembled on a DC linear motor (E-861, PI, German). The force range and resolution of force sensor are 14.1 N and 2.93 mN, respectively. The loading force and displacement during the mechanical test can be synchronously collected by the force sensor and linear motor, respectively.

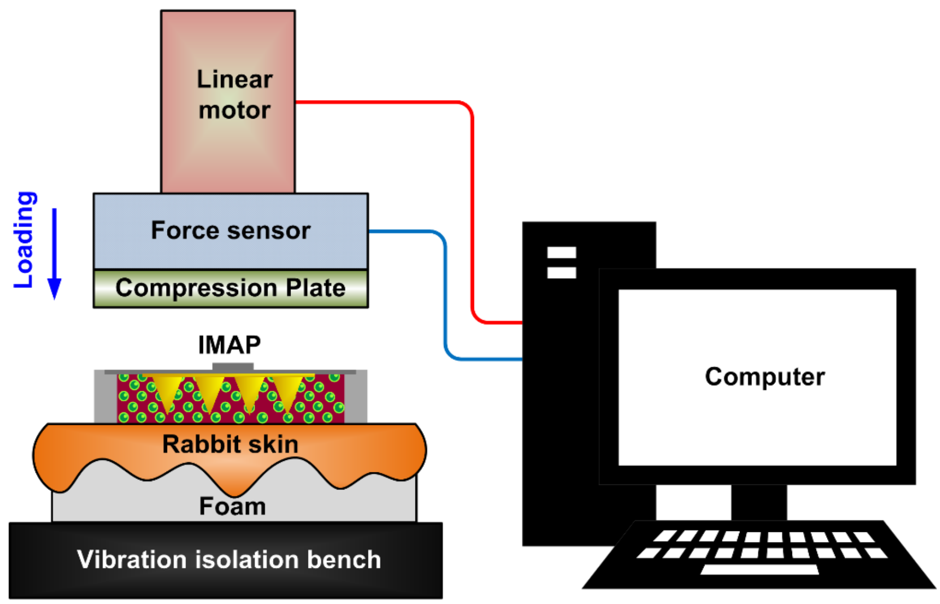

Figure S4 Schematic illustration of mechanical loading setup

5. *In vitro* transdermal drug delivery test

In vitro transdermal delivery of FITC-insulin loaded in IMAP through rabbit skin was investigated. The setup consisted of IMAP, vertical Franz diffusion cells (TP-3A, Albert Tech., China), iontophoresis-driven circuit, and smartphone, as shown in Figure S5. The receptor chamber was fully filled with phosphate buffered saline (PBS, pH 7.4). The receptor was warmed at 37 ± 1℃ and magnetically stirred at 300 rpm. The permeation area of a diffusion cell was about 201 mm^2. A fresh rabbit skin taped with an IMAP was assembled on a diffusion cell with the dermis contacting with PBS solution in the receptor chamber. Drug-loaded IMAP was employed as the upper donor chamber. The IMAP taped on the rabbit skin and Ag/AgCl electrode inserted in the PBS of receptor chamber were employed as the anode and cathode of iontophoresis, respectively. The smartphone powered iontophoresis-driven circuit. The iontophoresis-driven circuit promoted the transdermal delivery of liquid drug stored in IMAP into the PBS solution in receptor chamber of Franz diffusion cells.

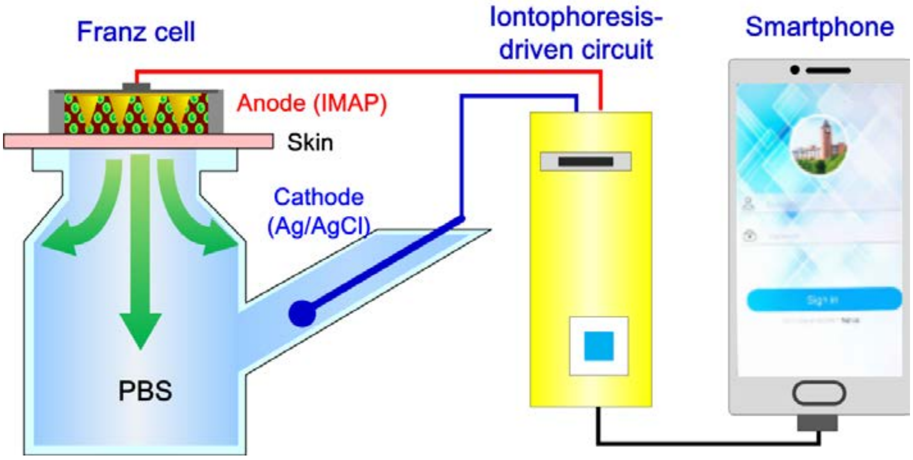

Figure S5 Schematic illustration of *in vitro* transdermal drug delivery setup

IMAPs under different usage conditions were divided into seven groups, as illustrated in Figure S6. (1) vesicles group: IMAP filled with 150 μL FITC-insulin loaded vesicles without application of compression and iontophoresis, (2) Insulin/MA/1 mA group: IMAP filled with 150 μL free insulin under 10 N compression and 1 mA iontophoresis, (3) vesicles/MA group: IMAP filled with 150 μL FITC-insulin loaded vesicles under 10 N compression without iontophoresis, (4) vesicles/1 mA group: IMAP filled with 150 μL FITC-insulin loaded vesicles under 1 mA iontophoresis, (5) vesicles/MA/1 mA group: IMAP filled with 150 μL FITC-insulin loaded vesicles under 10 N compression and 1 mA iontophoresis, (6) vesicles/MA/2 mA group: IMAP filled with 150 μL FITC-insulin loaded vesicles under 10 N compression and 2 mA iontophoresis, and (7) vesicles/MA/3 mA group: IMAP filled with 150 μL FITC-insulin loaded vesicles under 10 N compression and 3 mA iontophoresis.

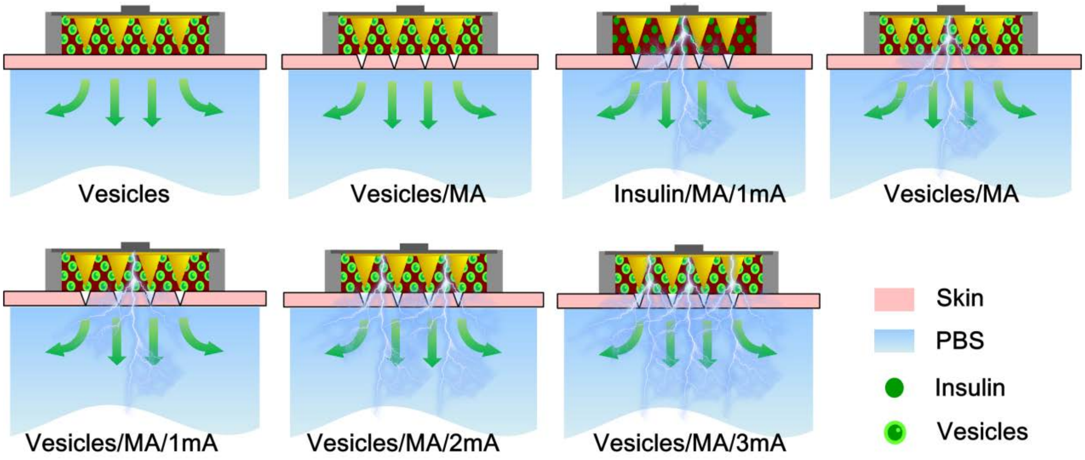

Figure S6 Schematic illustration of seven groups of IMAPs under different usage conditions

6. The specific parameters of IMAP components

IMAP is mainly composed of medical tape, anti-seepage gasket, medical sponge and solid MA.

The specific parameters of IMAP components are listed in Table S1. The components of IMAP are simple, easy-obtained and low cost. The total cost of IMAP is less than 0.16 dollar.

Table S1 The specific parameters of IMAP components

Components	Materials	Size	Price (USD)
Medical tape	PU film with adhesive	Diameter: Φ 40 mm;<br>Thickness: 0.05 mm.	< 0.04
Anti-seepage gasket	EVA foam	Inner circle diameter: Φ15mm;<br>Outer circle diameter: Φ22 mm;<br>Thickness: 1.1 mm.	< 0.02
Medical sponge	Polyvinyl alcohol	Porosity > 95 %;<br>Diameter: 15 mm;<br>Thickness: 1.1 mm.	< 0.02
Solid MA	PMMA	Microneedle number: 225;<br>Substrate length: 12 mm;<br>Microneedle height: 0.7 mm;<br>Microneedle base diameter: 0.18 mm.	< 0.08

7. The Mechanical performance

On “Press stage”, once the increasing stress reaches the rupture limit of skin, the microneedle tips penetrate into skin, resulting in a sudden drop of force at point ‘P’, as shown in Figure S7a. The critical penetration force at point ‘P’ is 1.6 N. On “Release stage”, once the microneedles are detached from the skin owing to the elastic rebound energy of IMAP, the friction force becomes zero, thereby resulting in an increase of the measured resistance force at point ‘Q’, as shown in Figure S7b. The resistance force at point ‘Q’ is approximately 1 N.

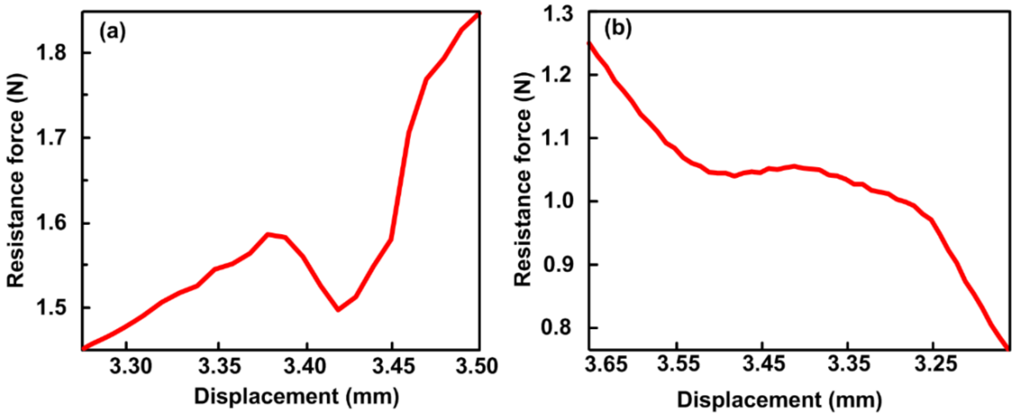

Figure S7 (a) The relationship between the resistance force and the loading displacement of the point ‘P’ on “Press stage”. (b) The relationship between the resistance force and the loading displacement of the

point 'Q' on "Release stage".

8. *In vitro* transdermal insulin delivery performance

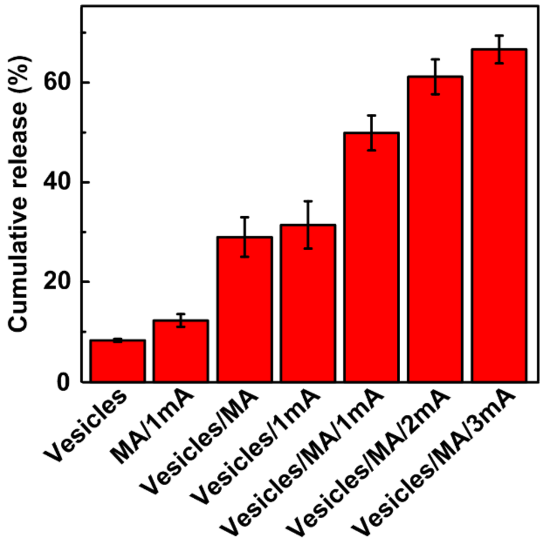

Figure S8 In vitro percentage of total cumulative amount release profiles of FITC-insulin from the reservoir into phosphate buffer within 5 h, including vesicles group, vesicles/MA group, insulin/MA/1mA group, vesicles/1mA group, vesicles/MA/1mA group, vesicles/MA/2mA group and vesicles/MA/3mA group.

The percentage of total cumulative amount of FITC-insulin released from the reservoirs was approximately 8.42±0.01 %, 12.44±0.01 %, 29.16±0.04 %, 31.51±0.05 %, 55.05±0.04 %, 61.28±0.03 % and 66.78±0.03 % for Vesicles, Vesicles/MA, Insulin/MA/1mA, Vesicles/1mA, Vesicles /MA/1mA, Vesicles /MA/2mA and Vesicles /MA/3mA groups, respectively.

The 3D confocal reconstruction and their different cross-section images of rabbit skins treated by vesicles/1mA group, vesicles/ MA group, and vesicles/MA/1mA group for one-hour are shown in Figure S9(a-c), respectively. As shown in these resulting pictures, IMAP integrates solid MA with iontophoresis and charged nanovesicles can achieve a synergistic and remarkable enhancement in transdermal delivery.

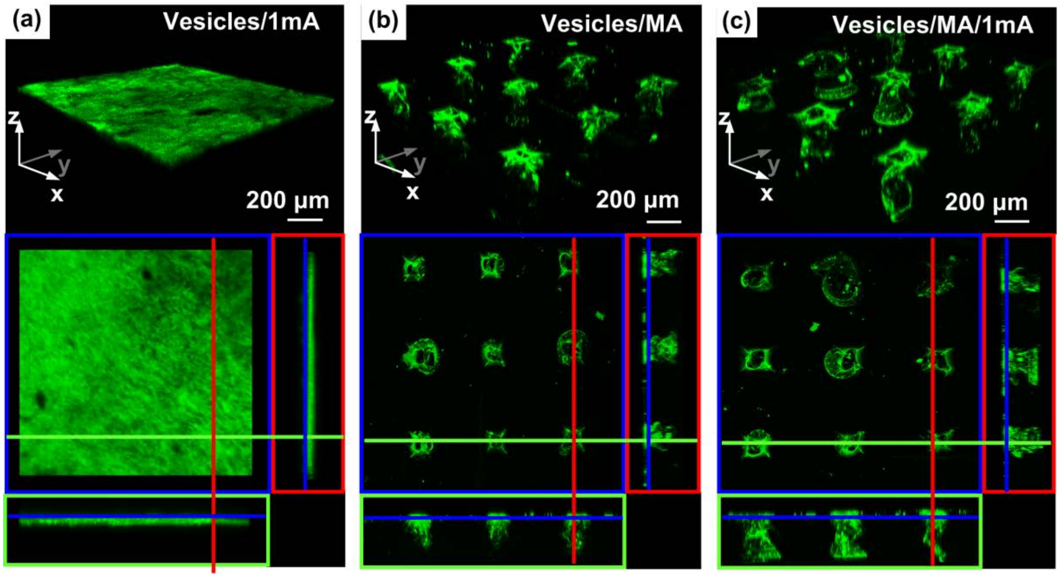

Figure S9 Confocal micrographs and their 3D reconstruction images of rabbit skins treated by (a) vesicles/1mA group, (b) vesicles/ MA group, and (c) vesicles/MA/1mA group.

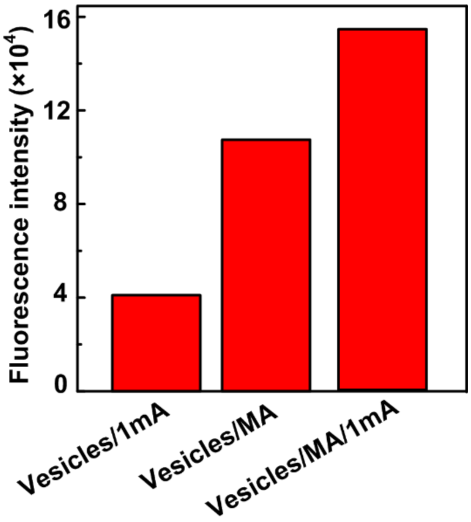

Figure S10 The total fluorescence intensity of the 3D confocal reconstruction images of rabbit skins treated by vesicles/1mA, vesicles/MA and vesicles/MA/1mA group.

After a series of numerical integration and multiplication from the fluorescence intensity data, the total fluorescence intensity of vesicles/1mA group, vesicles/MA group and vesicles/MA/1mA group are 41021.1, 107500.9 and 151413.4, respectively. The permeation amount of insulin in the skin is further quantified, demonstrating the excellent synergistic and remarkable enhancement of IMAP.

9. Ethics statement

All animal procedures conducted in this work were reviewed, approved, and supervised by the

Institutional Animal Care and Use Committee (IACUC) at the Sun Yat-Sen University (Approval Number: IACUC–DD–16–0904).

10. *In vivo* transdermal insulin delivery in diabetic rats

The diabetic rats whose BGL is in the range of 100-200 mg/dL is regarded in the normoglycemic state. The effective period that the diabetic rats are in normoglycemic states is defined as the normoglycemic time. Thus, the normoglycemic time of injection group is only 2.3 h because of the hypoglycemia. The normoglycemic time of diabetic rats in vesicles/MA/1mA group (6.8 h) is approximately 3.1-fold and 1.2-fold of that in injection group and vesicles/MA group (5.7 h), respectively. It implies the amalgamation strategy of MA with iontophoresis and nanovesicles can decrease the time lag, thereby accelerating the therapy via transdermal delivery without the risk of hypoglycemia. A more perspicuous statistical graph of the three group (vesicles/MA/1mA group, injection group and vesicles/MA group) and their detailed BGLs measured by blood glucose meter are shown in Figure S11-S12.

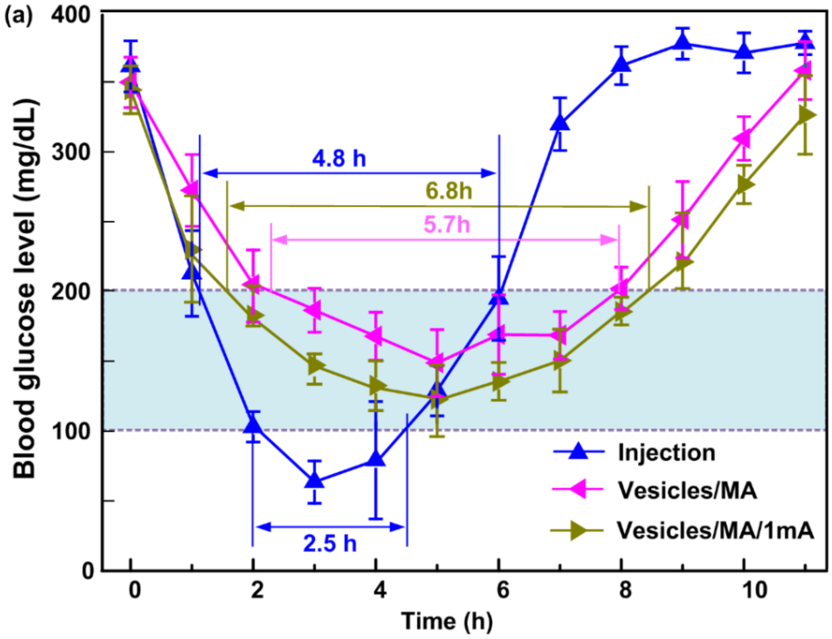

Figure S11 BGLs of diabetic rats of each group administrated with injection group, vesicles/1mA group and vesicles/MA/1mA group. The results are expressed as the mean ± standard deviation.

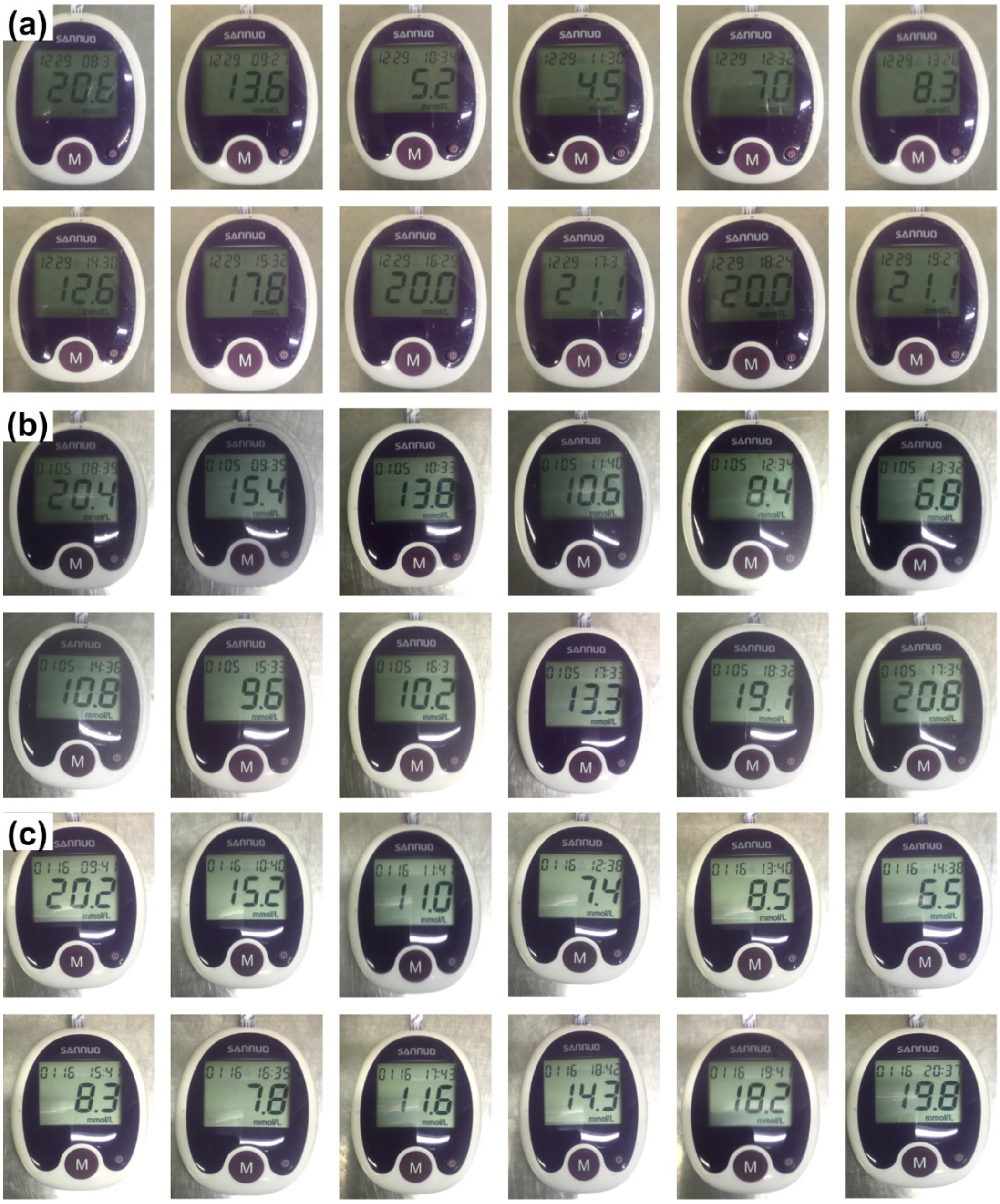

Figure S12 The real-time BGLs measured by blood glucose meter of diabetic rats of (a) injection group, (b) vesicles/1mA group and (c) vesicles/MA/1mA group. The unit of measurement by using blood glucose meter is 'mmol/L' which can be converted to '18 mg/dL' (i.e., 1 mmol/L = 18 mg/dL)

11. Supplementary Video

Video S1: The smartphone-based drug delivery system and the transdermal drug delivery mechanism of IMAP
